# Development of an echo‐shifted, multi‐echo, gradient‐echo sequence for T_2_ * quantification of slow‐relaxing water pools

**DOI:** 10.1002/mrm.30624

**Published:** 2025-07-04

**Authors:** Seonyeong Shin, Ana‐Maria Oros‐Peusquens, Seong Dae Yun, Ezequiel Farrher, N. Jon Shah

**Affiliations:** ^1^ Institute of Neuroscience and Medicine 4, INM‐4, Forschungszentrum Jülich Jülich Germany; ^2^ RWTH Aachen University Aachen Germany; ^3^ Institute of Neuroscience and Medicine 11, INM‐11, JARA, Forschungszentrum Jülich Jülich Germany; ^4^ JARA ‐ BRAIN ‐ Translational Medicine Aachen Germany; ^5^ Department of Neurology RWTH Aachen University Aachen Germany

**Keywords:** diffusivity, echo‐shifting technique, multi‐echo gradient echo, quantitative parameter mapping, T_2_* relaxation time

## Abstract

**Purpose:**

Although conventional multi‐echo gradient‐echo (GRE) sequences effectively quantify short and intermediate T_2_* in brain tissue, and general interest in cerebrospinal fluid (CSF) is growing due to its association with the glymphatic system, quantifying T_2_* in CSF remains underexplored. Accurate quantification of the slow‐relaxing water pools requires imaging at long echo times, significantly increasing acquisition time. This study proposes a novel sequence capable of quantifying the entire range of T_2_* without prolonged acquisition time, mapping T_2_* in both CSF and brain tissue.

**Methods:**

The proposed echo‐shifted, multi‐echo GRE (ES‐mGRE) combines the conventional multi‐echo GRE sequence with an echo‐shifting technique. Additional gradients are introduced, producing echoes in the next sub–repetition time interval.

**Results:**

ES‐mGRE generates artifact‐free images at both short and long echo times without extending acquisition time. Increasing the area of the additional gradients enhances diffusion sensitivity, allowing simultaneous quantification of T_2_* and *D* in CSF. The mean T_2_* of white matter and gray matter is 55.9 ms and 51.5 ms at 3 T, respectively. The mean T_2_* in the ventricles is 234.5 ms. The simultaneously quantified mean *D* value of 3.07 μm^2^/ms is closely aligned with the reference diffusivity.

**Conclusion:**

We demonstrate that the proposed ES‐mGRE sequence can effectively quantify the T_2_* of both CSF and brain tissue while also providing simultaneous diffusion information.

## 1 INTRODUCTION

Cerebrospinal fluid (CSF) is clinically significant in the diagnosis of various diseases and is routinely analyzed to detect biomarkers for brain conditions such as infections or inflammations of the central nervous system. Recently, CSF has also been used for liquid biopsies in glioma diagnosis.^1^ Lumbar puncture remains the typical method used for this procedure.

Interest in CSF has grown due to its role in the glymphatic system, which is hypothesized to function as a brain waste clearance system.^2^, ^3^, ^4^ Quantitative MRI (qMRI) enables the noninvasive quantification of tissue properties such as T_1_, T_2_, T_2_*, diffusion, or flow, which may serve as biomarkers for brain disease and have been the focus of recent studies.^5^, ^6^, ^7^, ^8^

The T_2_* relaxation time can be measured, for example, using the quantitative T_2_* image (QUTE) sequence,^9^, ^10^, ^11^ a variant of the multi‐echo gradient‐echo (mGRE) sequence. QUTE obtains multiple echoes via a bipolar readout and uses a multislice acquisition method, facilitating accurate T_2_* mapping with whole‐brain coverage. The in‐house‐developed mGRE sequence was originally reported in Shah et al.^9^ and Dierkes et al.^10^ under the acronym QUTE (quantitative T_2_* image). We have continued to use this acronym in several related publications^11^, ^12^, ^13^, ^14^, ^15^; however, because the sequence is a multi‐echo gradient echo implementation with bipolar readout, herein we will use, in most instances, the terms “in‐house‐implemented mGRE” and “echo‐shifted mGRE” instead of the acronyms QUTE and ES‐QUTE, which were used when reporting our preliminary results.^16^, ^17^

Although mGRE sequences efficiently measure T_2_* in brain parenchyma, research relating to T_2_* in CSF remains limited. If T_2_* in CSF can be shown to correlate with glymphatic clearance, it could be further explored in relation to neurodegenerative diseases.

**FIGURE 1:**
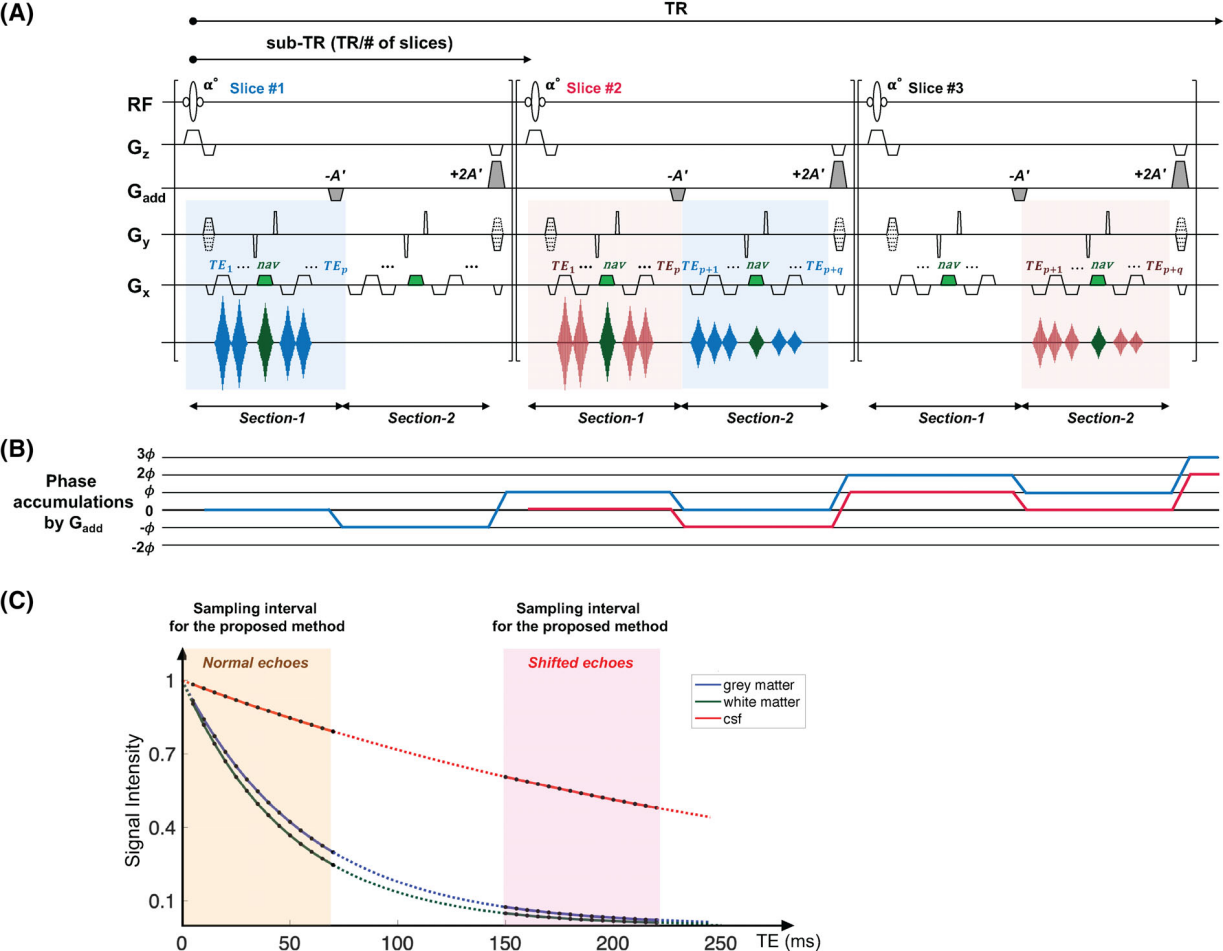
(A) Schematic diagram of the proposed echo‐shifted, multi‐echo gradient echo. (B) Corresponding phase plot. (C) Signal decay of gray matter, white matter, and cerebrospinal fluid (CSF). The colored areas indicate the time intervals during which the signals are sampled by the proposed method. The interval in between, where the expected signal decay is indicated with a dotted line, is not sampled. RF, radiofrequency; TE, echo time; TR, repetition time.

While the number of echoes used for T_2_* mapping is important for the fit performance, the time interval spanned by these echoes needs to extend to at least T_2_* for each tissue type for optimal performance.^11^ Gray and white matter have relatively comparable T_2_* at 3 T^9^, ^10^, ^11^; however, T_2_* values in CSF are an order of magnitude longer compared with brain tissue, requiring a much longer interval of TE values for similar performance of quantification. A long maximum TE is generally accompanied by an increase in TR, which in turn prolongs the acquisition time. Although the acquisition time can be reduced by using, for example, parallel‐imaging techniques, acceleration is constrained by the coil configuration and required signal‐to‐noise ratio (SNR).^18^, ^19^ The main drawback of attempting to quantify the T_2_* of both brain parenchyma and CSF with comparable accuracy is that a large fraction of the measurement time is spent acquiring echoes that do not contribute to the characterization of the already decayed brain‐tissue signal (Figure 1C).

Moonen et al.^20^ first proposed an echo‐shifting technique in which spins excited by a radiofrequency (RF) pulse are refocused in the subsequent repetition‐time (TR) interval via additional gradients, enabling the acquisition of long echo‐time (TE) images within a minimal scan time. However, RF pulses in subsequent TR intervals might interfere with the signal of interest.^21^ To mitigate this, echo‐shifting techniques are often paired with multislice acquisition methods.^22^, ^23^

In this work, the echo‐shifting technique was extended to the mGRE sequence for T_2_* quantification of both CSF and brain tissue. The proposed sequence, echo‐shifted multi‐echo GRE (ES‐mGRE) makes use of multislice acquisition to acquire both short‐TE and long‐TE images without extending scan time. In this work, ES‐mGRE was validated at 3 T using phantom and in vivo experiments, and the results demonstrate close alignment with the reference methods.

## 2 THEORY

Figure 1A shows a schematic diagram of the proposed sequence. ES‐mGRE combines an mGRE sequence^9^, ^10^, ^11^ with an echo‐shifting technique. In the following, a detailed description of the gradient characteristics is provided.

1. Readout and additional gradients: To acquire multiple echo data, a gradient reversal process is repeated as in conventional mGRE. A number of echoes, *p*, are first obtained. Both polarities of the readout gradients are used to obtain a larger number of echoes with a shorter echo‐spacing time than would be the case with a typical monopolar readout. Following the acquisition of *p* echoes, spins excited by *n*th RF pulse are then dephased with the first *additional* gradient (−A′). The dephased spins are rephased after the second gradient (+2A′) and the first additional gradient *in the next sub‐TR interval*. A number of echoes, *q*, are subsequently collected at long TEs. Spins excited by the *(n + 1)*th RF pulse in the next slice do not contribute to the echo‐shifted signals, as they are already dephased by the first additional gradient. This procedure is iterated. As a result, the readout period is separated into two parts: Section 1 collects the normal echoes, and Section 2 acquires the shifted echoes from the previous slice. The aim of this is to quantify short, intermediate, and long T_2_* by sampling the front and rear parts of the signal decay. After collecting (*p + q*) echoes, the readout dephasing gradient is duplicated at the end of each sub‐TR interval. If the total number of echoes, (*p + q*), is an even number, gradients with the same strengths but opposite polarities are employed.

The additional echo‐shifting gradients can, theoretically, be introduced along any axis. In addition, these gradients serve as diffusion‐weighting gradients, potentially enabling the measurement of diffusion properties.

2 Phase‐encoding gradients: A phase‐encoding gradient is applied at the beginning of each TR, and the gradient strength is incremented to cover the k‐space. Motion due to physiological processes, such as respiration or pulsation, is known to induce field variations in tissue, which can result in image artifacts and distort parameter quantification.^24^, ^25^ To minimize the effects of such field fluctuations, the acquisition of navigator echoes is incorporated and used for postprocessing corrections (see area in green). A phase rewinding and a winding gradient are applied before and after the navigator echo, respectively, within the shortest possible time. Following the acquisition of (*p* + *q*) echoes, the phase‐encoding gradient is rewound.
3 Slice‐selection gradients: A slice‐selection and a slice‐refocusing gradient are used at the beginning of each TR. The refocusing gradient is duplicated following the acquisition period.

Figure 1B shows the plots of phase accumulation induced by the additional gradients. The net phase becomes zero at the location of the echo read out. After the acquisition of shifted echoes, the spins excited by *n*th RF pulse are gradually spoiled by additional gradients in the subsequent sub‐TR intervals.

## 3 METHODS

### 3.1 Computer simulations

Computer simulations were performed to investigate the effect of echo‐shifting on T_2_* quantification and to optimize imaging parameters. A detailed explanation of the computer simulations and parameter setting are provided in the Supporting Information. Because ES‐ mGRE uses a multislice acquisition method with a single RF pulse per slice and effective TR (TR for both prompt and shifted echoes), the signal equation is equivalent to that of a spoiled GRE. This is in contrast to the signal loss that occurs in echo shifting with a single slice excitation, which involves multiple RF pulses per effective TR.^23^ Thus, the signal decay over time was adapted from that of spoiled GRE:

(1) $$\begin{array}{ll} & s\left({\mathrm{TE}}_{n}\right)=M_{0}\left(\frac{\sin\left(\alpha \right)\left(1-e^{-\mathrm{TR}/T_{1}}\right)}{1-\cos\left(\alpha \right)e^{-\mathrm{TR}/T_{1}}}\right)e^{-TE_{n}/T_{2}^{*}}e^{1i\left(\phi_{0}+2\pi ∆f{\mathrm{TE}}_{n}\right)}\\ & \;\times e^{-b\left(TE_{n}\right)D}+\varepsilon \left(0,\sigma^{2}\right), \end{array}$$

where *s*(TE_*n*_) represents the signal obtained at the *n*th TE; M_0_ is the spin density; and *α* is the flip angle. T_1_ and T_2_* are the longitudinal and effective transverse relaxation times, respectively; *ϕ*_0_ is an initial phase; ∆*f* is the off‐resonance frequency; *b*(TE_*n*_) is the diffusion‐weighting *b*‐value induced by additional gradients; and *D* represents the diffusion coefficient of each compartment. *ε*(0, *σ* ^2^) refers to additive white Gaussian noise.

Data sets consisting of 24, 32, and 64 echoes were created, each with *K* values of 4, 8, 10, 12, and 16. The value of *K* describes the area of the first additional gradient, set to *K* times that of the slice rephasing gradient. To determine how many echoes need to be shifted to provide reliable results, the number of shifted echoes was varied from 0 to *N*, the number of total echoes. The produced data sets were fitted as described in Section 3.5.

### 3.2 Phantom experiments

Phantom experiments were performed on a 3T MR scanner (MAGNETOM Prisma; Siemens Healthineers, Erlangen, Germany). A total of 12 data sets (i.e., 24, 32 and 64 echoes × *K* of 4, 8, 10, and 12) were obtained with the following imaging parameters: field of view (FOV) = 240 × 216 mm^2^, matrix size = 240 × 216, slice thickness = 2 mm, TR = 1200 ms, flip angle = 75°, pixel bandwidth = 800 Hz/px, TE_1_ = 3 ms, ΔTE = 1.47 ms, ΔPE = 0.8 ms (the time required for phase rewinding and winding gradients), and direction of additional gradients = slice selection. One‐third of the echoes were shifted to the next sub‐TR interval. Two navigator echoes were acquired in the middle of the normal and shifted echo train, respectively. The number of slices, *b*‐values, and TEs of the shifted echoes for each data set are described in Table S1.

Reference acquisitions of T_2_* and *D* values were performed using in‐house‐implemented mGRE, QUTE,^9^, ^10^, ^11^ and the manufacturer's spin‐echo, diffusion‐weighted, echo‐planar imaging (DW‐EPI), respectively. The QUTE data sets were acquired with the same imaging parameters as the ES‐mGRE, but with double the TR and number of echoes: TR = 2400 ms, number of echoes = 157 (including four navigator echoes located at Echoes 20, 59, 98, and 137). The DW‐EPI data sets were obtained with FOV = 240 × 216 mm^2^, matrix size = 240 × 216, slice thickness = 2 mm, TR = 5000 ms, number of slices = 24, pixel bandwidth = 1096 Hz/px, TE = 90 ms, GRAPPA acceleration factor = 2, partial Fourier factor = 6/8, and *b*‐value = 0, 20, 30, 40, 60, 80, 100, 200, 400, 600, 800, and 1000 s/mm^2^. The reference *D* values were also restricted to the slice‐selection direction, *D*_z_, with all the *b*‐values considered for the calculation.

### 3.3 In vivo experiments

In vivo data sets were obtained from 4 healthy volunteers. All subjects provided written, informed consent before the study. Imaging parameters were configured as in the first column of Table 1. The in‐plane resolution was kept at 1 × 1 mm^2^. The phase FOV (left–right direction) was adjusted for the head of each subject to minimize the scan time. Forty‐three of 64 echoes were prompt, and 21 shifted to the next sub‐TR interval, thereby ensuring sufficient sampling of the signal decay from both brain tissue and CSF. The TEs of the shifted echoes ranged from 206.88 to 237.72 ms, and the *b*‐values were about 180 s/mm^2^, corresponding to a value of *K* = 10, optimized according to computer simulation and phantom experiments.

**TABLE 1:** Imaging parameters for in vivo experiments.

	ES‐mGRE	In‐house‐implemented mGRE, QUTE
TR	3000 ms	
Flip angle	60°	
FOV read	240 mm	
In‐plane resolution	1 × 1 mm^2^	
Slice thickness	2 mm	
Bandwidth	800 Hz/px	
# of slices	23	11
# of echoes	64	157
# of navigators	2	4
(location)	(16th, 49th)	(20th, 59th, 98th, 137th)
# of shifted echoes	21	‐
*K* (*b*‐value of the shifted echoes)	10 (≈ 250 s/mm^2^)	‐
TEs	TE_1_ = 3 ms TE_43_ = 66.18 ms TE_44_ = 206.88 ms TE_64_ = 237.72 ms	TE_1_ = 3 ms TE_157_ = 238.08 ms
∆TE	1.47 ms	
∆PE	0.8 ms	

Abbreviations: ES, mGRE, echo‐shifted multi‐echo gradient echo; FOV, field of view; PE, phase encoding; QUTE, quantitative T_2_* image; TE, echo time; TR, repetition time.

For comparison, data sets were also obtained using in‐house‐implemented mGRE, QUTE.^9^, ^10^, ^11^ Imaging parameters that primarily affect SNR (e.g., TR, flip angle, bandwidth) were the same as those for ES‐mGRE but with an increased number of echoes to cover a similar TE range (second column). The vendor‐implemented Siemens mGRE sequence, which only allows for the acquisition of 12 echoes, was configured to have the same TE range as the unshifted echoes in ES‐mGRE, in‐plane resolution = 1.1 × 1.1 mm^2^, TE_1_ = 3 ms, ∆TE = 5.8 ms. The reference diffusivity values were acquired using DW‐EPI. A three‐dimensional (3D) magnetization‐prepared rapid gradient‐echo sequence was included for brain tissue segmentation (Table S2).

### 3.4 Postprocessing

Navigator echo correction was performed to compensate for field variations caused by physiological motion or, possibly, system drift from scanner instability. The correction method outlined in Refs. ^24^, ^25^ was modified for our study. Due to the very long TE interval and echo train used in our experiments, we found it necessary to acquire more than one navigator echo. This allows one to correct for variations occurring between phase‐encoding steps and during the echo train.

Two navigator echoes were acquired for ES‐mGRE: one in the middle of the unshifted echoes (*N*_u_) and the other in the middle of the shifted echoes (*N*_s_). The correction of ES‐mGRE data sets consists of two steps. First, a one‐dimensional (1D) Fourier transform was applied to all readouts. In the first step, the phase differences between the first and *p*th phase‐encoding steps were calculated using the phase of the first navigator echo. The 1D Fourier‐transformed k‐space lines of each echo, shifted and unshifted, as well as of later navigator echoes, were then corrected by multiplying the phase differences with the TEs, as follows:

(2a) $$s_{p}^{c}\left(x,k_{y},{\mathrm{TE}}_{n}\right)=s_{p}\left(x,k_{y},{\mathrm{TE}}_{n}\right)e^{-i\frac{\phi_{N_{\mathrm{up}}}\left(x,k_{y}\right)-\phi_{N_{u1}}\left(x,k_{y}\right)}{{\mathrm{TE}}_{\mathrm{nav}1}}\cdot {\mathrm{TE}}_{n}},$$

where *s*_*p*_^c^(*x*, *k*_*y*_, TE_n_) and *s*_*p*_ (*x*, *k*_*y*_, TE_n_) are the 1D Fourier‐transformed k‐space signals after and before the correction, respectively; TE_*nav*1_ is the TE of the first navigator echo; and ϕ_Nup_ (*x*,*k*_*y*_) – ϕ_Nu1_ (*x*,*k*_*y*_) represents the phase difference calculated between the first navigator of k‐space line *p* and the first navigator of k‐space line 1. In the second step, we calculate the phase differences involving the second navigator echoes, which have already been corrected by the first navigator as in Eq. (2a). The differences were then used to correct only the shifted echoes as follows:

(2b) $$\begin{array}{ll} & s_{p}^{c}\left(x,k_{y},{\mathrm{TE}}_{n}\right)=s_{p}\left(x,k_{y},{\mathrm{TE}}_{n}\right)e^{-i\frac{\phi_{N_{\mathrm{sp}}}^{c}\left(x,k_{y}\right)-\phi_{N_{s1}}^{c}\left(x,k_{y}\right)}{{\mathrm{TE}}_{\mathrm{nav}2}}\cdot {\mathrm{TE}}_{n}},\;\\ & \;\text{only for shifted echoes}. \end{array}$$

For the in‐house‐implemented mGRE‐QUTE acquisition, which uses more echoes to cover the same TE interval, four navigators were acquired. Each navigator was used to phase‐correct the closet echoes in the second step. For example, in a 128‐echo acquisition with four navigators, Echoes 1–32 were corrected by the first navigator only, Echoes 33–64 by the first and second navigators (Eqs. [2a] and [2b]), Echoes 65–96 by the first and third navigators, and Echoes 97–128 by the first and fourth navigators.

### 3.5 Fitting and analysis

The corrected k‐space data of individual coils were Fourier‐transformed and combined using an adaptive combine method.^26^ As stated in Section 2, using additional gradients adds a diffusion‐weighting effect to the images. Thus, T_2_* quantification was performed and compared without and with consideration of the diffusion effects.

(3) $$s\left({\mathrm{TE}}_{n}\right)=M_{0}e^{-TE_{n}/T_{2}^{*}}e^{-b\left(TE_{n}\right)\cdot D}e^{{\left(-1\right)}^{n}\delta }.$$

Equation (3) is an adaptation of Eq. (1) relevant to T_2_* fitting. Here, the diffusion weighting (*b*‐factor) and the bipolar readout (δ modulation) are directly included, whereas steady‐state effects (T_1_, flip angle) are included in the signal intensity at TE = 0 ms. The *b*‐values were computed from all imaging gradients except for the phase‐encoding gradients.^27^ As the data sets were acquired using bipolar readouts, amplitude modulation δ was considered^28^ and corrected with an iterative fitting, similar to that described in Refs. ^29^ and ^30^ by finding the value that minimizes the differences between acquired and synthesized images generated without δ. In computer simulations, δ was excluded, as the data sets were generated under conditions ideal for focusing only on the echo‐shifting gradient effects. For in‐house‐implemented mGRE, QUTE, and for the vendor‐implemented Siemens mGRE data sets, the diffusion term was neglected.

When the signal predominantly contains noise, which is often the case for long TEs, the fit of the magnitude signal can lead to an overestimation of T_2_*.^31^, ^32^ To avoid this bias, Rician noise distribution was incorporated in the fitting process.^33^ The noise variance was determined based on four ROIs positioned at the corners of the background, each consisting of 400 voxels.

To assess the accuracy and precision, the mean and standard deviation of the calculated T_2_* and *D* were measured. In the case of in vivo experiments, segmentation results obtained using *FreeSurfer* were used as masks.^34^, ^35^

## 4 RESULTS

**FIGURE 2:**
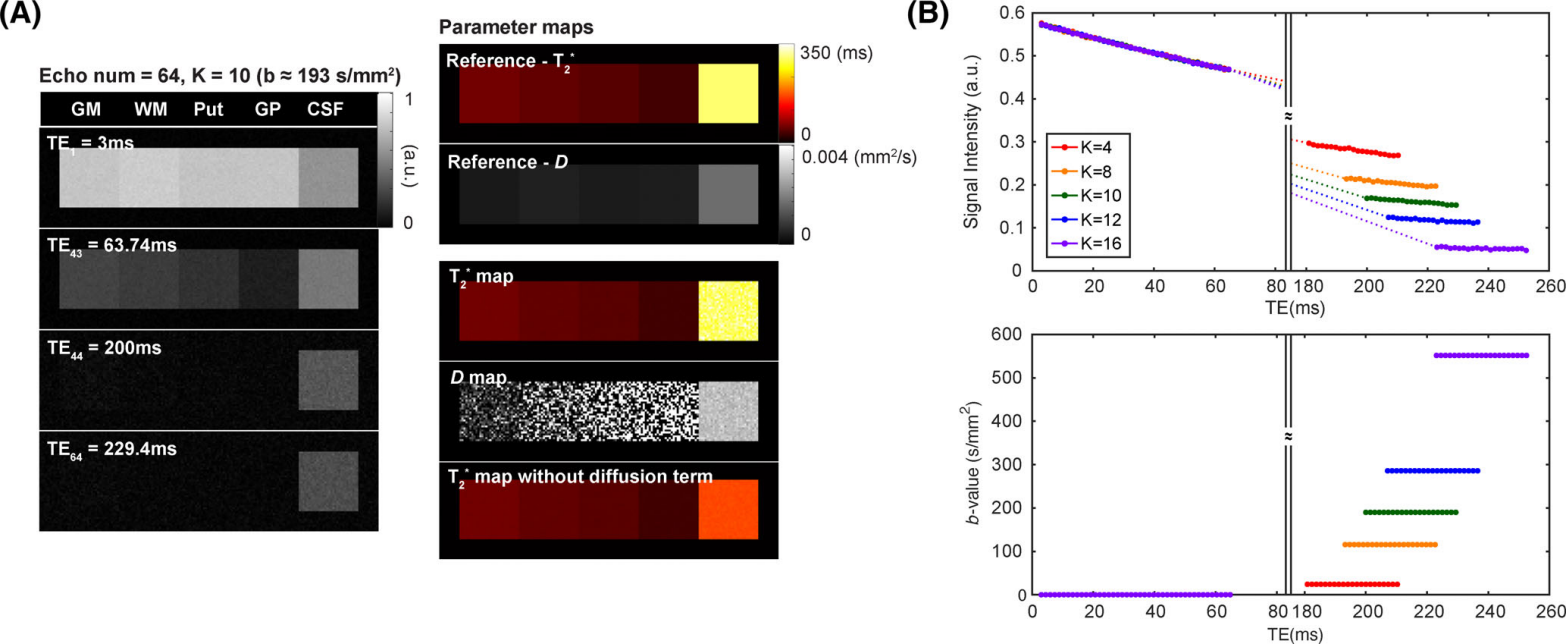
(A) Simulation data sets generated with 64 echoes and *K* equal to 10. One‐third of the total echoes were shifted. Each small rectangular region illustrates the expected behavior of different brain regions: gray matter (GM), white matter (WM), putamen (Put), globus pallidus (GP), and cerebrospinal fluid (CSF). (B) Plots of the signal decay versus echo time (TE) and *b*‐value versus TE in the cerebrospinal fluid (CSF) region for 64 echoes and various values of *K*. The parameter *K* scales the integral of the echo‐shifting gradients, thereby modulating diffusion sensitivity and signal reduction of the shifted echoes.

Figure 2 shows the results of computer simulations. Figure 2A displays the images for the different TE values, with the third and fourth rows corresponding to the shifted echo images. The quantified parameter maps are presented alongside the reference maps. The signal decay of brain tissue can be sufficiently sampled during the acquisition of the unshifted echoes. Because of its long TE values, only the signal from CSF remains in the shifted echo images. Incorporating the effect of diffusion into the signal model produces T_2_* values that are closer to the ground‐truth values. For the brain tissue with relatively low diffusivity, the quantified T_2_* values remained consistent, regardless of whether the diffusion was included in the model. Figure 2B displays plots of the signal decay and *b*‐values versus TE in the simulated CSF region when the number of echoes was set to 64. Each color represents a different area of the additional gradients, and thus a different diffusion weighting. As the area of additional gradients (*K*) increases, the signal intensity of the shifted echoes decreases and the shifted TEs increase, as described in the Computer Simulations section in the Supporting Information. More detailed simulation results demonstrating the effects of the number of shifted echoes and *K* are also provided in Figures S1 and S2. To summarize, the simulations suggest that shifting one‐third of the echoes with a sufficiently large area of the additional gradients (e.g., *b* ≈ 200 s/mm^2^ for 64 echoes) optimizes the quantification results, with diffusion being quantified for CSF.

**FIGURE 3:**
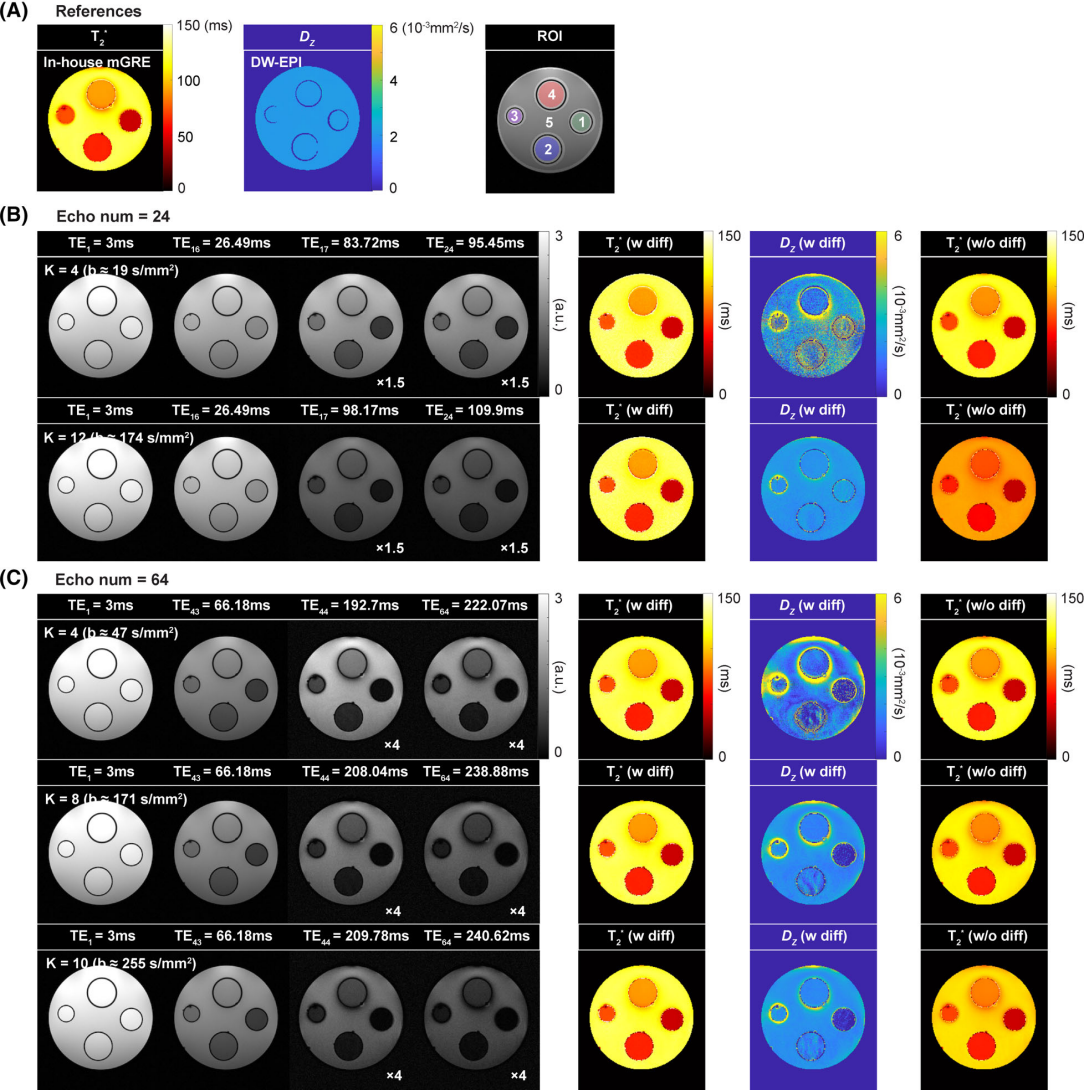
Results of phantom experiments obtained from reference (A) and data sets having 24 (B) and 64 (C) echoes. In (B) and (C), the first two columns show the normal (i.e., unshifted) echo images, whereas the third and fourth columns show the shifted echo images. The fifth to seventh columns depict the calculated quantitative maps with and without considering the diffusion term, respectively. Each row represents the results for different areas of additional gradients. The results with the different number of echoes and *K* can be found in Figure S3. DW‐EPI, diffusion‐weighted echo‐planar imaging; ROI, region of interest; TE, echo time.

Figure 3 displays the results of the phantom experiments. The proposed ES‐mGRE sequence produced echo images at very long TE values without significant artifacts. The signal intensity of the shifted echoes decreased as the *K* values increased, in line with the theoretical prediction. The calculated T_2_* and *D*_z_ maps exhibited the same trends as the computer simulations. Without consideration of the diffusion effect, biased T_2_* values were obtained. These values further deviated from the reference when *K* increased, especially in regions with relatively long T_2_*. The inclusion of the diffusion term in the fit allowed for *D*_z_ and T_2_* to be quantified, the latter with high accuracy. Increasing the area of the additional gradients improved the precision of *D*_z_. Reducing the number of echoes required a larger area of additional gradients for similar precision. Table 2 lists the mean and standard deviation of T_2_* and *D*_z_ within each ROI, calculated from the data sets with 64 echoes and *K* equal 10. The quantified values closely matched the reference values.

**TABLE 2:** Mean and standard deviation of the calculated T_2_*s and *D*_*z*_ for each region of interest (ROI) using data acquired with 64 echoes and *K* of 10 (21 shifted echoes with echo time starting from ˜210 ms).

	ROI 1	ROI 2	ROI 3	ROI 4	ROI 5
T_2_* (ms)	44.6 ± 0.7	63.1 ± 1.4	72.7 ± 1.7	90.0 ± 1.6	121.9 ± 3.2
*D*_*z*_ (μm^2^/ms)	0.5 ± 0.4	1.7 ± 0.6	2.2 ± 0.6	1.8 ± 0.2	2.0 ± 0.2
Reference (In‐house‐implemented mGRE, QUTE) ‐ T_2_*	44.5 ± 0.4	63.3 ± 0.6	72.8 ± 1.1	91.1 ± 0.6	122.6 ± 2.7
Reference (DW‐EPI) ‐ *D*_z_	2.1 ± 0.02				

Abbreviations: DW‐EPI, diffusion‐weighted echo‐planar imaging; mGRE, multi‐echo gradient echo; QUTE, quantitative T_2_* image.

**FIGURE 4:**
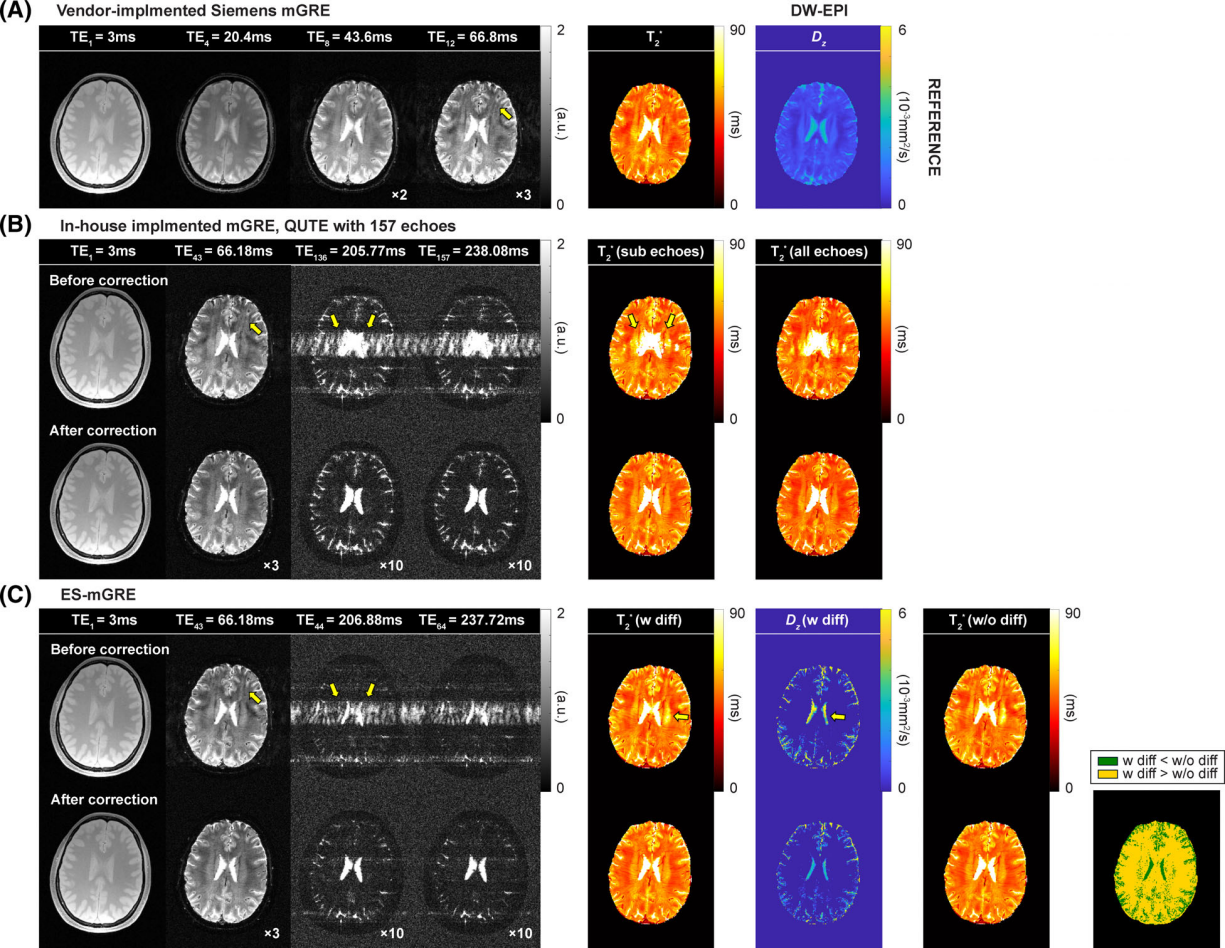
Results of in vivo experiments obtained using vendor‐implemented Siemens multi‐echo gradient echo (mGRE) (A), in‐house‐implemented mGRE (B), quantitative T_2_* image, and echo‐shifted, multi‐echo GRE (ES‐mGRE) sequences (C). In (B) and (C), the first and second rows depict the results before and after applying the navigator echo correction, respectively. The first to fourth columns display the acquired images. The yellow arrows indicate the ghost artifacts induced by physiological fluctuations. The derived quantitative maps are presented next to the images. For the in‐house‐implemented mGRE data sets, the T_2_* values were quantified after retrospectively selecting the images to match the echo time (TE) values of ES‐mGRE (*fifth column*), as well as considering all acquired TE images (*sixth column*). In ES‐mGRE, a map that compares corrected Akaike Information Criterion (cAIC) values is also displayed. The areas where the signal model with a diffusion term provides a better fit compared to the model without a diffusion term are indicated by the color green. Otherwise, it is represented by the yellow. DW‐EPI, diffusion‐weighted echo‐planar imaging.

Figure 4 shows the results from in vivo experiments. In images produced by the standard reconstruction software of the scanner, artifacts from CSF at the ventricles were present across the entire brain region and became more pronounced in the long TE images. Overlap of the actual signal and artifacts alters the signal intensity, reducing the accuracy of the quantified metrics. However, these artifacts were eliminated with navigator echo correction, showing that navigators are imperative for accurate mapping of long T_2_*, or imaging at long TEs. Following visual inspection of the navigator‐corrected T_2_* maps, ES‐mGRE offers clearer delineation of brain structures, such as the centrum semiovale, compared with the vendor‐implemented Siemens GRE, which does not acquire navigation echoes. Conversely, the T_2_* map from ES‐mGRE data is similar to that of the reference method (i.e., QUTE with subechoes). The T_2_* maps, with and without consideration of the diffusion term, are visually comparable. The last column in Figure 4C shows the map of corrected Akaike Information Criterion (cAIC) values, comparing the two fit methods.^36^, ^37^ Note that the cAIC values suggest that the incorporation of the diffusion term leads to a more accurate representation of the signal, particularly in CSF regions characterized by high diffusivity values. The method thus produces high‐resolution, distortion‐free diffusion maps of CSF.

**FIGURE 5:**
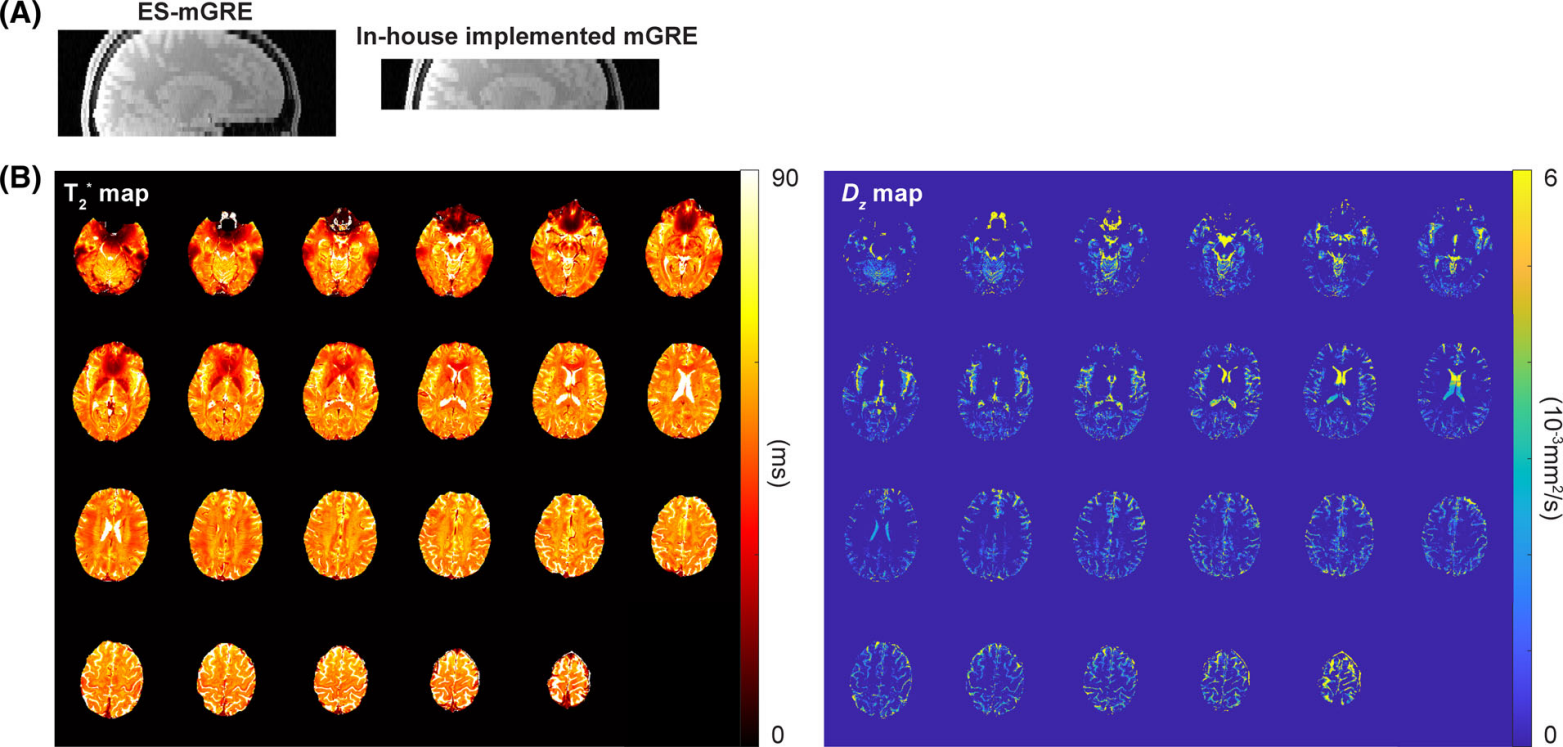
(A) Brain coverage of in‐house‐implemented multi‐echo gradient echo (mGRE) and echo‐shifted mGRE (ES‐mGRE). (B) Whole‐brain T_2_* and *D*_*z*_ maps obtained from ES‐mGRE.

Figure 5A illustrates the brain coverage of in‐house‐implemented mGRE and ES‐mGRE. The ES‐mGRE enables the acquisition of twice the number of slices compared with the mGRE within the same measurement time. Figure 5B presents comprehensive T_2_* and *D*_z_ maps of the whole brain obtained from ES‐mGRE. Note that, in particular, it is possible to quantify the diffusivity values of CSF not only within the ventricles but also within the subarachnoid space with high resolution. Table 3 lists the statistical measures of the calculated T_2_* and *D*_z_ values compared with the reference methods. The mean T_2_* values of gray and white matter agree with those obtained from the retrospectively subsampled mGRE. The median value for CSF T_2_* was consistent with that from mGRE. In addition, the ES‐mGRE‐derived *D*_z_ values were comparable to the values obtained by reference diffusion measurements. Across subjects, the average of the median T_2_* values in the lateral ventricles was 276.5 ms, substantially higher than in the third (192.7 ms) and fourth ventricles (231 ms), and the subarachnoid space (114.3 ms). All pairwise comparisons between the lateral ventricles and other regions showed statically significant differences (*p* < 0.0027; Kolmogorov–Smirnov test). Similarly, the mean *D*_z_ value in the subarachnoid space was lower than that in the lateral ventricle across all subjects, with all comparisons yielding *p*‐values < 7e‐28 (Kolmogorov–Smirnov test).

**TABLE 3:** Mean and standard deviation of the calculated T_2_*s and *D*_*z*_ from a single volunteer (*upper*) and across 4 subjects (*lower*).

	GM	WM	Third ventricle	Fourth ventricle	Lateral ventricles	Subarachnoid space
T_2_* (ms)	54.6 ± 11	50.1 ± 6.6	216.7 (137.7, 479.9)	276.4 (120.5, 521.3)	304.2 (143.3, 786)	119.9 (93.7, 186.1)
T_2_* (without diffusion)	54.5 ± 11	49.9 ± 6.5	105.4 (95.8, 112.5)	106.7 (93.7, 110.8)	133.8 (109.7, 181.4)	102.4 (89.6, 124.0)
Reference (in‐house‐implemented mGRE, QUTE) ‐ T_2_*	52.7 ± 10.1	47.8 ± 6.4	‐	‐	319.7 (165.6, 648.1)	118.7 (94.4, 174.5)
Reference (in‐house‐implemented mGRE, QUTE with subechoes) ‐ T_2_*	55.2 ± 10.3	49.9 ± 6.3	‐	‐	302.1 (159.7, 606.7)	115.5 (93.5, 162.5)
*D*_z_ (μm^2^/ms)			3.13 (2.7, 4.5)	3.32 (2.8, 4.3)	3.3 (2.8, 4.1)	3.01 (2.5, 3.8)
Reference (DW‐EPI) ‐*D*_z_			3.1 (2.4, 3.7)	3.47 (3.14, 3.7)	2.96 (2.69, 3.2)	2.5 (2.23, 2.83)
Average values across subjects						
T_2_*	55.9 ± 1.8	51.5 ± 2.2	192.7 ± 29.4	231 ± 101.3	276.5 ± 42.3	114.3 ± 3.14
*D*_z_			3.44 ± 0.4	3.54 ± 0.3	3.37 ± 0.5	3.02 ± 0.4

*Note*: The median, lower quartile (Q1), and upper quartile (Q3) were calculated for the cerebrospinal fluid regions (80 ≤ T_2_* ≤ 2000 ms and 0.002 ≤ *D*_z_ ≤0.005 mm^2^/s), given the fact that the distribution of values is not Gaussian. In the case of in‐house‐implemented multi‐echo gradient echo, the information from the third and fourth ventricles was not obtained due to its limited slice coverage.

Abbreviations: DW‐EPI, diffusion‐weighted echo‐planar imaging; GM, gray matter; mGRE, multi‐echo gradient echo; QUTE, quantitative T_2_* image; WM, white matter.

## 5 DISCUSSION

In this study, an echo‐shifting technique was combined with mGRE, an established method for high‐quality T_2_* mapping,^9^, ^10^, ^11^ for the quantification of T_2_* of CSF with high accuracy and precision. The use of additional gradients facilitated the acquisition of T_2_*‐weighted images at long TEs without significantly increasing the scan time. The scan time is only prolonged by sub‐TR (TR/no. of slices), which has a negligible duration of around 100 ms for the performed acquisition with 64 echoes. In our design, two additional gradients with an area ratio of −1:2 were periodically applied to shift the echoes. In principle, a single additional gradient could be used in an alternating manner (i.e., −A for one sub‐TR and A for the next sub‐TR). Using fewer additional gradients might reduce the influence of eddy currents. However, the signals are balanced in such a way that the spins excited from *n*th RF pulse would be rephased again after two sub‐TR intervals and interfere with the signal from another slice. In the proposed sequence design, the signals from *n*th RF pulse are gradually dephased over time after acquiring shifted echoes (Figure 1B). ES‐mGRE adopts an interleaved acquisition strategy, which circumvents the signal loss commonly associated with traditional echo‐shifting techniques, where two pulses contribute to the signal from one slice.^22^, ^23^ The area of additional gradients needs to be considerable; otherwise, there is still a risk of signal contamination from previous slices. Optimization is required, as an excessive increase in the area of additional gradients can lead to a longer scan time, necessitating a reduction in the number of echoes or slices obtainable within a given TR.

From the signal‐fitting perspective, the lowest standard deviation of T_2_* is achieved with a continuous and unshifted echo train. However, such an acquisition requires an increased scan time. Furthermore, covering an extensive range of TE does not necessarily provide additional information about the signal decay of tissue (Figure 1C). ES‐mGRE addresses these limitations by selectively acquiring both short and long TE images within a shorter scan time, providing a practical balance between accuracy and efficiency.

Incorporating a diffusion term, exp.(−*b*⋅*D*), is crucial for the accurate quantification of T_2_* with ES‐mGRE, as the signal loss between unshifted and shifted echoes is influenced by the diffusion‐weighting effect of the echo‐shifting gradients. However, depending on the tissue properties and sequence parameters, the inclusion of the diffusion term may introduce instability in the fit and reduce the precision of the quantification, as shown by the simulation results (Figure S2). The slightly increased standard deviation of T_2_* for ES‐mGRE compared with in‐house‐implemented mGRE primarily arises due to this added complexity in the fit. Nevertheless, despite this minor trade‐off, it enables simultaneous mapping of T_2_* and diffusivity of CSF with high accuracy.

In principle, increasing the area of the additional gradients magnifies the difference in the intensity of the unshifted versus the shifted echo signals, which aids in determining T_2_* and diffusivity values. However, for tissues with short T_2_* and/or large diffusivity values, excessively large gradients may overly suppress the intensity of the shifted echoes. Thus, setting appropriate imaging parameters is crucial for accurate and precise quantification and will depend on the characteristics of the tissue properties to be quantified. The use of 64 echoes (21 shifted) produced accurate and precise T_2_* mapping for brain tissue and demonstrated good accuracy and precision in the T_2_* and *D* mapping for CSF, as shown both by simulations and experimental comparison with standard measurements.

The use of computer simulations allowed for the optimization of imaging parameters and the selection of the signal model. Although opting for a mono‐exponential fitting without the diffusion term might compromise the accuracy of the quantification of T_2_*, this approach enhances its precision, as the number of free variables is reduced. When the additional gradients have a small area, and the number of shifted echoes is less than one‐third of the total echoes, the discrepancy in the quantified T_2_* values—with or without the inclusion of a diffusion term—is negligible, particularly in low diffusivity regions, such as brain tissue. Conversely, when using larger additional gradients, fitting without the diffusion term yields a marked deviation from the true T_2_* values. This deviation can be quantified from simulations for given values or ranges of T_2_* and diffusivity, as shown in this work. Thus, even without explicitly modeling the diffusion term, ES‐mGRE represents a valuable tool for intersubject or intrasubject comparison with high sensitivity. Alternatively, T_2_* can also be calculated using the *D* values predefined from other literature or sequences, thereby reducing the number of variables in a fit.

With the optimized settings used in this work, ES‐mGRE was able to obtain twice the number of slices compared with the in‐house‐implemented mGRE sequence within the same scan time. Alternatively, mGRE requires doubled acquisition time to achieve the same number of slices. Therefore, in the time required for mGRE, several ES‐mGRE data sets can be obtained, such as with two optimized acquisition parameters to allow for adequate quantification in both tissue and CSF. Alternatively, because the *b*‐value range from the echo‐shifting gradients is relatively low (< 300 s/mm^2^), one could use several *b*‐values to investigate tissue perfusion using the intravoxel incoherent motion technique.^38^, ^39^

Notably, according to the spoiled GRE equation (Eq. [1]), the signal intensity increases nonlinearly with TR, and the additional signal gain resulting from increasing TR becomes marginal at longer TRs. For instance, doubling TR from 3000 ms to 6000 ms (assuming a T_1_ of 4300 ms for CSF and adjusting the corresponding Ernst angle) results in only a 34% increase in signal. The trade‐off, evaluated as SNR/acquisition time, is inefficient, given that doubling the scan time leads to less than twice SNR. This theoretical consideration emphasizes ES‐mGRE as a more practical and time‐efficient strategy for achieving greater spatial coverage without increasing TR.

Navigator echoes were acquired to correct for artifacts induced by physiological motion. Using navigator echoes from a relatively short TE allows for the precise estimation of resonance frequency (*f*0) variations. However, the *f*0 variation calculated from short TE navigators might not represent those present at longer TEs. Thus, the acquisition protocol was configured to obtain several navigator echoes along the echo train, separately for the unshifted and shifted echoes. However, using navigator echoes obtained at long TE poses a challenge due to the low SNR in brain parenchyma, which complicates the calculation of phase differences. Too low SNR at a specific position, *x*, causes spikes in the calculated phase difference and enhances instead of reducing artifacts in the images. For voxels with SNR values below average, the median value of the higher SNR regions was used to correct the global field variations.

**FIGURE 6:**
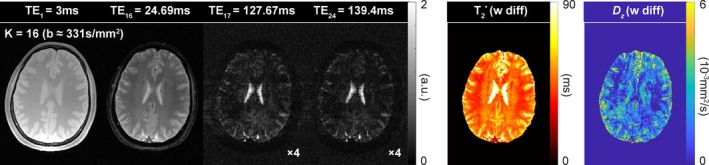
Acquired images and calculated quantitative maps when using the data sets with 24 echoes. TE, echo time.

The calculated *D*_*z*_ values in the phantom experiments matched the reference values in regions with relatively large *T*_2_* values when *K* was large. However, in regions with shorter T_2_* values, obtaining accurate values of *D*_*z*_ was unsuccessful, even at larger *K* values. This is because during the acquisition of the shifted echoes, the signals characterized by shorter T_2_* are already decayed. To accurately quantify the *D*_z_ values for the short T_2_* regions, an effective approach can be to reduce the number of echoes, thereby decreasing the sub‐TR and TEs of shifted echoes (Figure 3B). With a reduced number of echoes, ES‐mGRE can still provide reliable T_2_* quantification owing to its capability to acquire shifted echo images in the next sub‐TR interval and thus extend the T_2_* fitting range to an adequate TE interval. This applies to both phantom and in vivo data. Although diffusivity quantification in brain tissue and/or CSF remains a secondary benefit (Figure 6), the key advantage of this work lies in the robust and efficient T_2_* mapping that ES‐mGRE offers.

The quantified *D*_z_ of CSF in vivo depends on the location within the ventricles. The values were consistent and comparable to those reported in the literature for the central part of the ventricles but were found to be larger toward the anterior horn/lower part of the ventricles. A possible cause is that these areas exhibit a faster T_2_* decay, leading to less signal in the shifted echo images. A possible explanation of this observation is that the ventricles are composed of complex structures, including the highly irrigated choroid plexus and further blood vessels,^40^ and it is possible that susceptibility effects might contribute to the obtained values. Locally varying oxygenation of CSF could also affect T_2_*. A more likely cause for the increased *D*_*z*_ and decreased T_2_* is the presence of flow, but this is not currently included in the simulations or fit model. Recent studies have shown that D* from the intravoxel incoherent motion technique can reflect CSF flow.^41^, ^42^ In our work, *D*_*z*_ was calculated with relatively small *b*‐values. A simple diffusion term in our model, *D*_*z*_, while limited, can still partially provide information on CSF dynamics.

For most brain tissues with intermediate T_2_* and relatively low diffusivity, the diffusion coefficient is, at best, an estimate. However, if diffusivity and T_2_* are increased in brain tissue, for example, in the case of pathologies such as vasogenic edema ^38^ or white‐matter hyperintensities,^43^ ES‐mGRE *may offer the additional benefit* of simultaneous high‐resolution diffusion estimate, in addition to accurate T_2_* mapping in the affected regions. A natural application of our sequence is the investigation of long‐T_2_* free water in brain parenchyma, known to be associated with aging and/or neurodegeneration.^40^, ^44^, ^45^, ^46^ This can be done either by quantifying the intensity of very long echo images, or (better) by multicomponent evaluation of the signal.

It should be noted that *D*_*z*_ is not a rotationally invariant metric. In this regard, a more complete characterization of water diffusion could be obtained by combining measurements with different orientations of the echo‐shifting gradients. Although this would substantially increase the measurement time, the possibility of obtaining diffusion maps with perfect anatomical fidelity and high resolution is promising. A further possibility would be to adapt single‐shot trace diffusion‐weighting schemes^47^, ^48^ to our purposes. However, this is beyond the scope of the present work.

The present implementation of ES‐mGRE was designed to shift part of the echoes into the next sub‐TR interval. It is also feasible to acquire additional shifted echoes after two or more sub‐TR intervals with an appropriate sequence design. However, a limitation of an increasing number of echo intervals is that the TEs of shifted echoes are constrained by the total number of echoes and sub‐TR values, and thus, numerical optimization considering the T_2_* of targeted regions is required.

## 6 CONCLUSIONS

A novel ES‐mGRE sequence has been proposed in this study. By using additional gradients, both short and long TE images can be acquired without a substantial increase in acquisition time. The use of navigator echoes to correct for phase variations both between k‐space lines and along the echo train was found to be crucial for image and quantification quality. The obtained images allowed for simultaneous quantification of T_2_* and 1D diffusivity/flow in CSF.

## Supporting information


**Figure S1.** Reference (A) and calculated (B–D) T_2_* and *D* maps with respect to the number of echoes and areas of additional gradients. (B,C,D) Results from data sets with 24, 32, and 64 echoes, respectively, where one‐third of the echoes were shifted. The *K* and corresponding *b*‐values of the shifted echoes are written in each row.
**Figure S2.** Plots of the mean and standard deviation of the calculated T_2_*s and *D*s in each region of interest (ROI). (A–C) Data sets with 24, 32, and 64 echoes, respectively. In each panel, the first and second rows show the mean and standard deviation of the diffusion‐considered T_2_*s, whereas the third and fourth rows show the mean and standard deviation of the calculated *D*s. The mean and standard deviations of the T_2_*s when the diffusion effect was not considered are shown in the fifth and sixth rows. The x‐axis is the number of shifted echoes. Each color denotes the different area of the additional gradients.
**Figure S3.** Results of phantom experiments with respect to the number of echoes. (A–C) Images and quantitative parameter maps acquired from the data sets having 24, 32, and 64 echoes, respectively. In each panel, the fifth and sixth columns present the T_2_* and *D* maps, whereas the seventh column shows the T_2_* maps calculated without considering the diffusion term in a fit model.
**Table S1.** Imaging parameters for phantom experiments when the number of echoes was 24 (A), 32 (B), and 64 (C). The number of slices was set to be the maximum possible, while minimizing the time required for additional gradients.
**Table S2.** Imaging parameters for diffusion‐weighted echo‐planar imaging (DW‐EPI) and three‐dimensional magnetization‐prepared rapid gradient‐echo (MP‐RAGE) sequences used in in vivo experiments.
