# Supplementary material for: Development of an echo‐shifted, multi‐echo, gradient‐echo sequence for T2 * quantification of slow‐relaxing water pools
Source: Magn Reson Med. 2025 Jul 4;94(5):2057–70. doi: 10.1002/mrm.30624 (PMC12393191; doi:10.1002/mrm.30624)
Supplement: Supplementary file 1 — Figure S1. Reference (A) and calculated (B–D) T2* and D maps with respect to the number of echoes and areas of additional gradients. (B,C,D) Results from data sets with 24, 32, and 64 echoes, respectively, where one‐third of the echoes were shifted. The K and corresponding b‐values of the shifted echoes are written in each row. Figure S2. Plots of the mean and standard deviation of the calculated T2*s and Ds in each region of interest (ROI). (A–C) Data sets with 24, 32, and 64 echoes, respectively. In each panel, the first and second rows show the mean and standard deviation of the diffusion‐considered T2*s, whereas the third and fourth rows show the mean and standard deviation of the calculated Ds. The mean and standard deviations of the T2*s when the diffusion effect was not considered are shown in the fifth and sixth rows. The x‐axis is the number of shifted echoes. Each color denotes the different area of the additional gradients. Figure S3. Results of phantom experiments with respect to the number of echoes. (A–C) Images and quantitative parameter maps acquired from the data sets having 24, 32, and 64 echoes, respectively. In each panel, the fifth and sixth columns present the T2* and D maps, whereas the seventh column shows the T2* maps calculated without considering the diffusion term in a fit model. Table S1. Imaging parameters for phantom experiments when the number of echoes was 24 (A), 32 (B), and 64 (C). The number of slices was set to be the maximum possible, while minimizing the time required for additional gradients. Table S2. Imaging parameters for diffusion‐weighted echo‐planar imaging (DW‐EPI) and three‐dimensional magnetization‐prepared rapid gradient‐echo (MP‐RAGE) sequences used in in vivo experiments. [file MRM-94-2057-s001.docx]

**Supplementary Information**

***Computer simulations***: The numerical phantom was designed to reflect five brain components, namely (1) global grey matter (GM), (2) global white matter (WM), (3) putamen (Put), (4) globus pallidus (GP), and (5) CSF. Each of the five regions had a matrix size of 30×30.

Signal changes over time were produced based on the equation for a spoiled GRE.

$s\left( \mathrm{TE}_{n} \right)=M_{0}\left( \frac{\sin\left( \alpha\right)\left( 1-e^{-TR/T_{1}} \right)}{1-\cos\left( \alpha\right)e^{-TR/T_{1}}} \right)e^{-TE_{n}/T_{2}^{*}}e^{1i\left( \phi_{0}+2\pi\Delta f\mathrm{TE}_{n} \right)}e^{-b\left( TE_{n} \right)D}+\varepsilon\left( 0,\sigma^{2} \right)$, [1]

The TEs of ES-QUTE can be expressed as:

$\mathrm{TE}_{n}=\left\{ \begin{aligned} \mathrm{TE}_{1}+\left( n-1 \right)\Delta\mathrm{TE}, normal echo \\ \mathrm{TR}_{\mathrm{sub}}+T_{\mathrm{add}}+\mathrm{TE}_{1}+\left( n-1 \right)\Delta\mathrm{TE}, &shifted echo \end{aligned} \right.$ , [2]

where TE_1_ is the first echo time, and ∆TE is the echo spacing time. TR*_sub_* denotes the time between successive RF pulses and can be defined as TR*_sub_* =TR/(no. of slices). The term T_add_ is the time required for applying the first additional gradient (-A'). In Eq. [2], the small additional shift in echo times created by the phase rewinding and winding gradients required for the acquisition of navigator echoes was not considered.

Three parameters, namely the different number of echoes and slices (which interdepend, see below), the number of shifted echoes, and the area of additional gradients, were explored as they influence the T_2_^*^ quantification. We concentrate here on features that are specific to the echo-shifted multi-echo GRE sequence developed here; the usual signal variation of conventional mGRE is well described in the literature (1-3).

Most of the simulation parameters have concomitant effects on the quantification of T_2_^*^ and diffusion. For a given TR (i.e., restricted scan time), the maximum number of echoes that can be acquired is inversely proportional to the number of slices. As seen in Eq. [2], the variation in the number of slices also results in changes to the TR*_sub_*, subsequently affecting the TE of the shifted echoes. Another point to consider is that the echo-shifting gradients also function as diffusion-weighting gradients. The general equation for the *b*-value can be written as:

$b\left( TE_{n} \right)=\gamma^{2}\int_{0}^{TE_{n}} G^{2}dt$, [3]

where γ is the gyromagnetic ratio and G(t) is represented by

$G\left( t \right)=\int_{0}^{t} g\left( t^{'} \right)dt^{'}$. [4]

g(t') is the effective gradient strength from all imaging gradients. The *b*-value is indeed affected by the timing of the applied gradients. Thus, changes in TR*_sub_* resulting from the alterations of the number of slices also affect the *b*-value. Moreover, an increase in the area of additional gradients leads to an increase in the *b*-value, consequently enhancing the diffusion effects. Hence, simulation datasets were generated for different combinations of the number of echoes and the area of the additional gradients.

For the sake of convenience, M_0_ was fixed to unity for all five regions, *α* was set to 60°, and TR to 3000ms. The T_1_ and T_2_^*^ values in each region were assigned as follows: 1421 ms, 930 ms, 1337 ms, 1043 ms, and 4300 ms for T_1_, and 58 ms, 50 ms, 45 ms, 33 ms, and 300 ms for T_2_^*^ (4-8). The initial phase, *ϕ*_0_, was set to 0° and the offset frequency, ∆*f*, was specified to be 10 Hz. The TE_1_, and ∆TE were set to 3 ms and 1.47 ms, respectively. When the area of the first additional gradient (-A') was set to *K* times that of the slice rephasing gradients, T_add_ can be approximated as rounding off (867.1×*K*+115.1) μs from the Siemens programming platform (IDEA). The time for the second additional gradients, T_add2_, was approximated as rounding off (1734×*K* – 752.6) μs with an amplitude of 16 mT/m. To simplify the calculation of the *b*-value, only the additional gradients were considered to contribute and assumed to be rectangular pulses. The values of *D* for each region were set to 0.7, 0.89, 0.74, 0.82, and 3 (×10^-3^ mm^2^/s) (9,10). Gaussian noise was introduced to the signal so that the signal-to-noise ratio (SNR) of white matter in the first echo image was around 65, which is a typical value of our acquired data.

**Table S1.** Imaging parameters for phantom experiments when the number of echoes was (a) 24, (b) 32, and (c) 64. The number of slices was set to be the maximum possible, while minimizing the time required for additional gradients.

**a) 24 echoes**

| **K** | **4** | **8** | **10** | **12** |
| --- | --- | --- | --- | --- |
| # of slices | 23 | 19 | 17 | 16 |
| b-value | ≈19s/mm^2^ | ≈82s/mm^2^ | ≈120s/mm^2^ | ≈175s/mm^2^ |
| TEs of  unshifted echoes | TE_1_ = 3ms  TE_16_ = 26.49ms | | | |
| TEs of  shifted echoes | TE_17_ = 83.72ms  TE_24_ = 95.45ms | TE_17_ = 98.17ms  TE_24_ = 109.9ms | TE_17_ = 107.34ms  TE_24_ = 119.07ms | TE_17_ = 113.48ms  TE_24_ = 125.21ms |

**b) 32 echoes**

| **K** | **4** | **8** | **10** | **12** |
| --- | --- | --- | --- | --- |
| # of slices | 19 | 16 | 15 | 14 |
| b-value | ≈24s/mm^2^ | ≈90s/mm^2^ | ≈141s/mm^2^ | ≈208s/mm^2^ |
| TEs of  unshifted echoes | TE_1_ = 3ms  TE_22_ = 35.31ms | | | |
| TEs of  shifted echoes | TE_23_ = 103.52ms  TE_32_ = 118.19ms | TE_23_ = 118.83ms  TE_32_ = 133.5ms | TE_23_ = 125.57ms  TE_32_ = 140.24ms | TE_23_ = 133.02ms  TE_32_ = 147.69ms |

**c) 64 echoes**

| **K** | **4** | **8** | **10** | **12** |
| --- | --- | --- | --- | --- |
| # of slices | 10 | 9 | 9 | 9 |
| b-value | ≈47s/mm^2^ | ≈171s/mm^2^ | ≈255s/mm^2^ | ≈354s/mm^2^ |
| TEs of  unshifted echoes | TE_1_ = 3ms  TE_43_ = 66.18ms | | | |
| TEs of  shifted echoes | TE_44_ = 191.23ms  TE_64_ = 222.07ms | TE_44_ = 208.04ms  TE_64_ = 238.88ms | TE_44_ = 209.78ms  TE_64_ = 240.62ms | TE_44_ = 211.51ms  TE_64_ = 242.35ms |

**Table S2.** Imaging parameters for DW-EPI and 3D MP-RAGE sequences used in *in vivo* experiments.

|  | **DW-EPI** | **3D MP-RAGE** |
| --- | --- | --- |
| TR | 3400 ms | 2250 ms |
| Flip angle | 90° | 7° |
| Resolution | 1.2 × 1.2 × 2 mm^2^ | 0.94 × 0.94 × 0.94 mm^2^ |
| Bandwidth | 1096 Hz/px | 620 Hz/px |
| # of slices | 25 | 256 |
| Partial Fourier | 6/8 | 6/8 |
| Acceleration factor | 2 | 2 |
| TE | 73 ms | 1.79 ms |
| TI | - | 1000 ms |
| b-values (s/mm^2^) | 0, 20, 30, 40, 60, 80, 100, 200, 400, 600, 800, and 1000 | - |

Figure S1 shows the results of computer simulations for different numbers of echoes and areas of additional gradients. Without the diffusion term, an increase in the area of additional gradients, *K*, leads to a biased T_2_^*^ in CSF. Incorporating the effect of diffusion into the signal model produces T_2_^*^ values that are closer to the ground truth values. For the brain tissue with relatively low diffusivity, the quantified T_2_^*^ values remained consistent, regardless of the value of *K*. An increase in *K* leads to improved accuracy of *D* in the CSF. With a smaller number of echoes and larger values of K (i.e., 24 echoes with *K* of 16), it also becomes feasible to quantify *D* in simulated ROIs with properties corresponding to brain tissue. This will be explored in future experiments; in the present *in vivo* work, we concentrate on the properties of CSF. For CSF, the T_2_^*^ values calculated with 64 echoes are more accurate, and a larger area of additional gradients is required with a smaller number of echoes in order to achieve similar accuracy/precision in the quantification of *D*.

**Figure S1.** (a) Reference and (b-d) calculated T_2_^*^ and *D* maps with respect to the number of echoes and areas of additional gradients. Panels (b), (c) and (d) show the results from datasets with 24, 32, and 64 echoes, respectively, where one-third of the echoes were shifted. The *K* and corresponding *b*-values of the shifted echoes are written in each row.

Figure S2 illustrates plots of the mean and standard deviation of the measured values with respect to the number of shifted echoes and *K*. If the diffusion term was not considered, an increase in the number of shifted echoes and *K* resulted in a biased T_2_^*^. The bias was more pronounced as *K* increased, while an increase in the *K* led to a smaller standard deviation in the biased T_2_^*^. The standard deviation of T_2_^*^ displays a shallow minimum over a broad shifted-echo range. When diffusion was taken into account, the mean T_2_^*^ was initially close to the true value until approximately one-third of the echoes were shifted. The standard deviation of T_2_^*^ increased with the number of shifted echoes. The mean *D* approximated the true value well, provided that the number of shifted echoes was not too small. Increasing *K* reduced the standard deviation of *D*.

**Figure S2.** Plots of the mean and standard deviation of the calculated T_2_^*^s and *D*s in each ROI. Panels (a), (b) and (c) were obtained from datasets with 24, 32, and 64 echoes, respectively. In each panel, the first and second rows show the mean and standard deviation of the diffusion-considered T_2_^*^s, while the third and fourth rows show the mean and standard deviation of the calculated Ds. The mean and standard deviations of the T_2_^*^s when the diffusion effect was not considered are shown in the fifth and sixth rows. The x-axis is the number of shifted echoes. Each color denotes the different area of additional gradients.


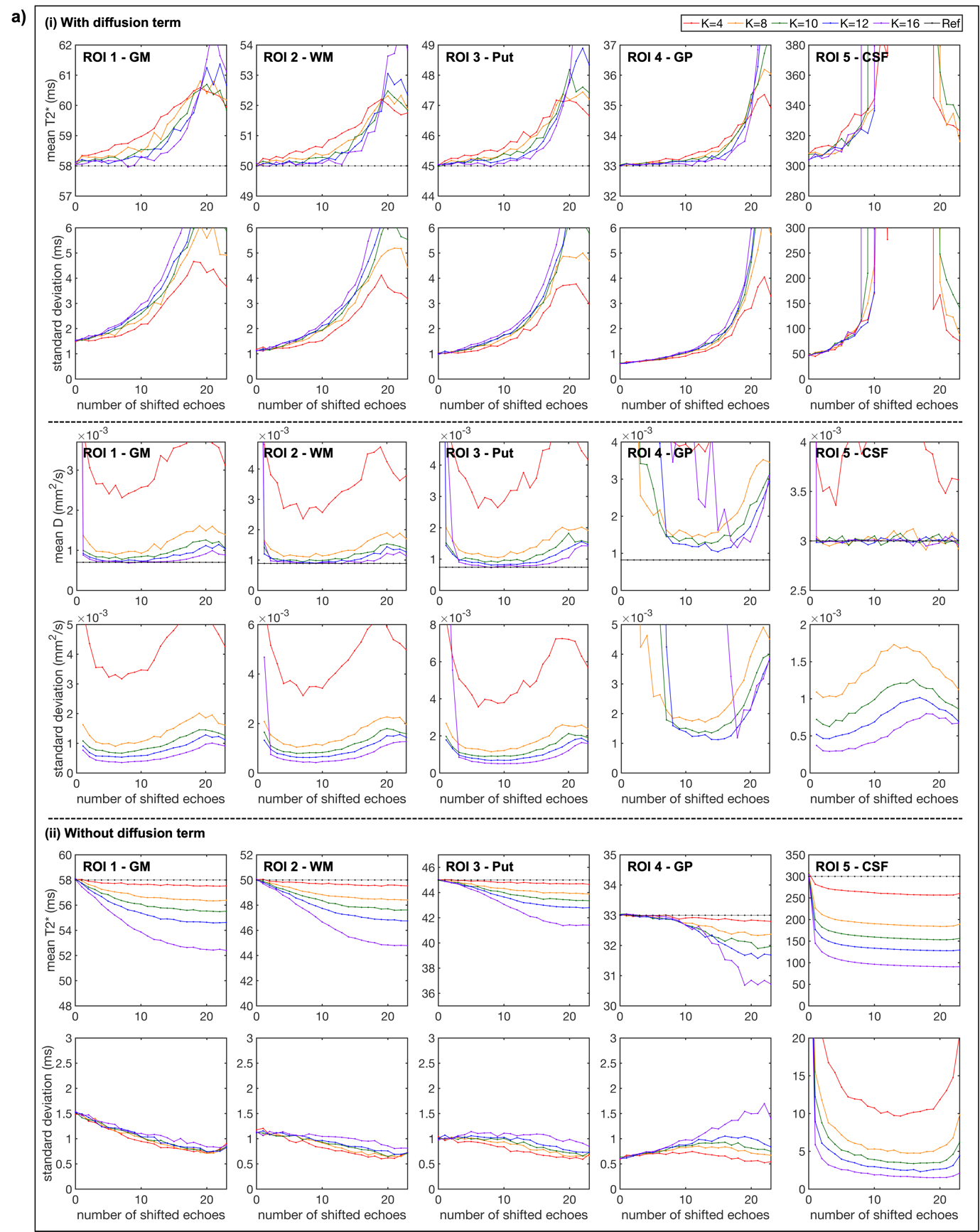


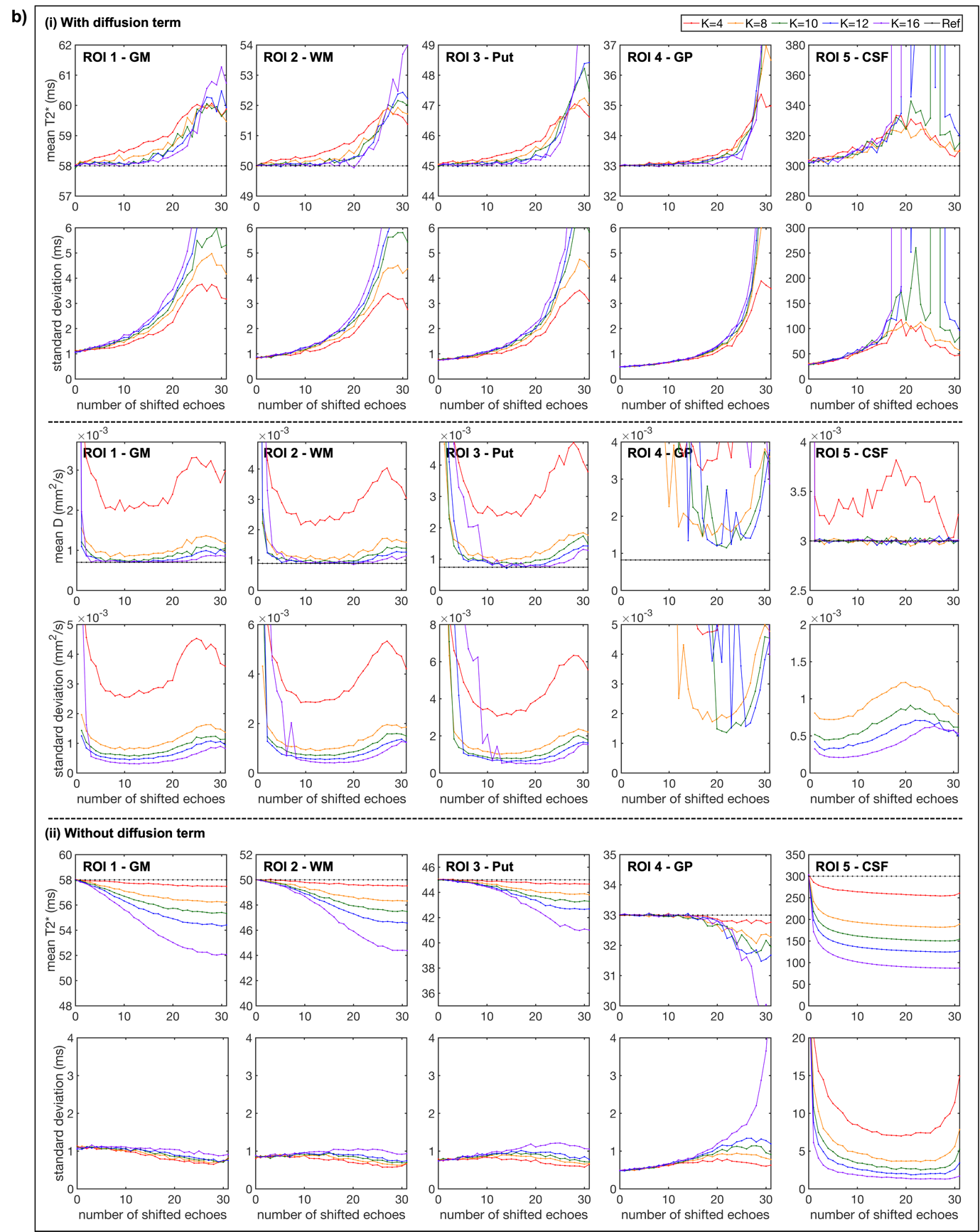


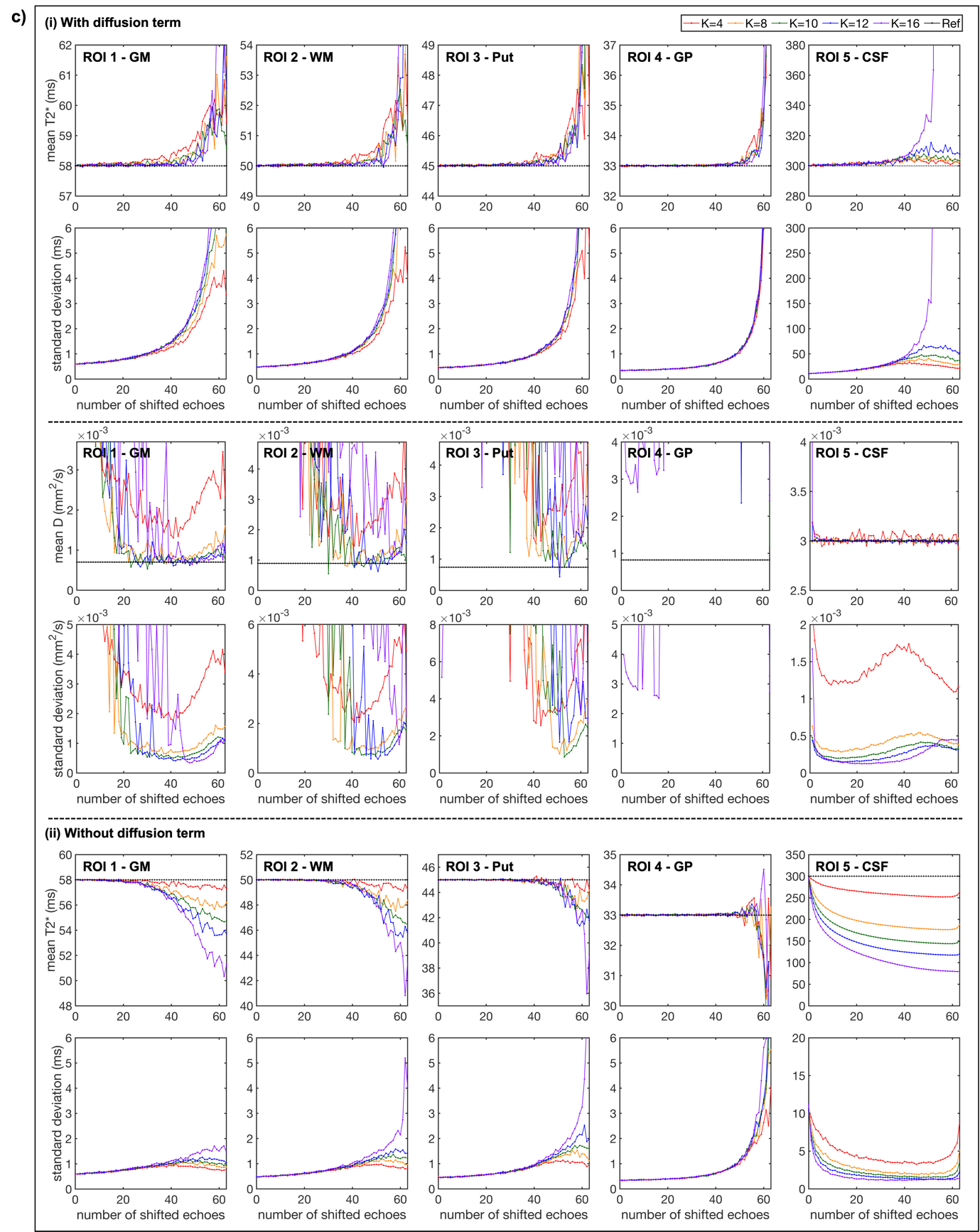


**Figure S3.** Results of phantom experiments with respect to the number of echoes. Panels (a), (b) and (c) show the images and quantitative parameter maps acquired from the datasets having 24, 32, and 64 echoes, respectively. In each panel, the fifth and sixth columns present the T_2_^*^ and *D* maps, while the seventh column shows the T_2_^*^ maps calculated without considering the diffusion term in a fit model.

**References**

[1] Dathe H, Helms G. Exact algebraization of the signal equation of spoiled gradient echo MRI. Phys Med Biol. 2010;55(15):4231-4245. doi:10.1088/0031-9155/55/15/003

[2] Helms G, Dechent P. Increased SNR and reduced distortions by averaging multiple gradient echo signals in 3D FLASH imaging of the human brain at 3T. J Magn Reson Imaging. 2009;29(1):198-204. doi:10.1002/jmri.21629

[3] Helms G, Dathe H, Dechent P. Quantitative FLASH MRI at 3T using a rational approximation of the Ernst equation [published correction appears in Magn Reson Med. 2010 Apr;63(4):1136]. Magn Reson Med. 2008;59(3):667-672. doi:10.1002/mrm.21542

[4] Zhu DC, Penn RD. Full-brain T1 mapping through inversion recovery fast spin echo imaging with time-efficient slice ordering. Magn Reson Med. 2005;54(3):725-731. doi:10.1002/mrm.20602

[5] Gelman N, Ewing JR, Gorell JM, Spickler EM, Solomon EG. Interregional variation of longitudinal relaxation rates in human brain at 3.0 T: relation to estimated iron and water contents. Magn Reson Med. 2001;45(1):71-79. doi:10.1002/1522-2594(200101)45:1<71::aid-mrm1011>3.0.co;2-2

[6] Yamashiro A, Kobayashi M, Saito T. Cerebrospinal fluid T1 value phantom reproduction at scan room temperature. J Appl Clin Med Phys. 2019;20(7):166-175.

doi:10.1002/acm2.12659

[7] Oros-Peusquens AM, Loução R, Abbas Z, Gras V, Zimmermann M, Shah NJ. A Single-Scan, Rapid Whole-Brain Protocol for Quantitative Water Content Mapping With Neurobiological Implications. Front Neurol. 2019;10:1333. Published 2019 Dec 20. doi:10.3389/fneur.2019.01333

[8] Rooney WD, Johnson G, Li X, et al. Magnetic field and tissue dependencies of human brain longitudinal 1H2O relaxation in vivo. Magn Reson Med. 2007;57(2):308-318. doi:10.1002/mrm.21122

[9] Helenius J, Soinne L, Perkiö J, et al. Diffusion-weighted MR imaging in normal human brains in various age groups. AJNR Am J Neuroradiol. 2002;23(2):194-199.

[10] Sener RN. Diffusion MRI: apparent diffusion coefficient (ADC) values in the normal brain and a classification of brain disorders based on ADC values. Comput Med Imaging Graph. 2001;25(4):299-326. doi:10.1016/s0895-6111(00)00083-5
